# Burn Injury Leads to Increase in Relative Abundance of Opportunistic Pathogens in the Rat Gastrointestinal Microbiome

**DOI:** 10.3389/fmicb.2017.01237

**Published:** 2017-07-06

**Authors:** Guangtao Huang, Kedai Sun, Supeng Yin, Bei Jiang, Yu Chen, Yali Gong, Yajie Chen, Zichen Yang, Jing Chen, Zhiqiang Yuan, Yizhi Peng

**Affiliations:** Institute of Burn Research, Southwest Hospital, State Key Laboratory of Trauma, Burns and Combined Injury, Third Military Medical UniversityChongqing, China

**Keywords:** gastrointestinal microbiota, 16S rRNA sequencing, burn, α-diversity, β-diversity

## Abstract

The gastrointestinal microbiome is crucial in human health. With greater than 10 times the cell count of an individual, the gastrointestinal microbiome provides many benefits to the host. It plays an important role in chronic illnesses and immune diseases and also following burns and trauma. This study aimed to determine whether severe burns affect the gastrointestinal microbiome during the early stages of after burn injury and the extent to which the microbiome is disturbed by such burns. We used a rat burn model to investigate any changes occurring in the microbiome after the burn trauma using 16S rRNA sequencing and downstream α-diversity, β-diversity, and taxonomy analysis. With 128631 and 143694 clean sequence reads, an average of 2287 and 2416 operational taxonomic units (OTUs) were recognized before and after the burn injury, respectively. Bacterial diversity within the pre- and post-burn groups was similar according to OTU richness, Chao 1 index, Shannon index and ACE index. However, the constituents of the gastrointestinal microbiota changed after the burn injury. Compared with the pre-burn samples, the post-burn samples showed a tendency to cluster together. The ratio of Firmicutes to Bacteroidetes decreased after the burn injury. Also, the abundance of some probiotic organisms (i.e., butyrate-producing bacteria and Lactobacillus) decreased after the burn injury. In contrast, opportunistic pathogenic bacteria, such as those of the genera *Escherichia* and *Shigella* and the phylum of Proteobacteria are more abundant post-burn. In conclusion, dysbiosis in the gastrointestinal microbiome was observed after the burn injury. Although the total number of species in the gastrointestinal microbiome did not differ significantly between the pre- and post-burn injury groups, the abundance of some bacterial components was affected to various extents.

## Introduction

Distinct from its traditional function in assisting the absorption of some nutrients, the gastrointestinal microbiome plays a role in immune and inflammatory responses (Fujimura et al., 2010; Sekirov et al., 2010; Sommer and Backhed, 2013). Dysbiosis of the healthy gastrointestinal microbiome is correlated with many chronic, immune-related diseases, such as diabetes, inflammatory bowel disease, various other autoimmune diseases, and cardiovascular disease (Sekirov et al., 2010; Human Microbiome Project Consortium, 2012b). For example, as an immunologically privileged organ, the eyes can be affected by specific T-cells activated in the gut, and this can eventually lead to autoimmune uveitis (Horai et al., 2015).

Beside their role in chronic illnesses and immune-related diseases, the function of the gastrointestinal microbiome in trauma and emergency onset diseases has also been uncovered (Sekirov et al., 2010; Harris et al., 2014; Kuethe et al., 2016). Sepsis caused by autogenous infections is still the main reason for mortality in burns patients. The gastrointestinal epithelial barrier is destroyed after burns, leading to the translocation of endotoxins and bacteria that are only supposed to exist in this tract (Earley et al., 2015). The healthy microbiome acts as a physiological barrier preventing opportunistic pathogen infections. Many Gram-positive bacteria in the gastrointestinal tract produce butyrate, which serves as a preferred energy source for colonocytes (Scheppach et al., 1995). Butyrate is produced mainly by Gram-positive bacteria belonging to the XlVa cluster of the Clostridium subphylum of low-G+C-content gram-positive bacteria (Collins et al., 1994; Willems et al., 1996; Barcenilla et al., 2000). Feedback between gastrointestinal microbiome and the immune system is important for establishing tolerance along gut mucosal surfaces and maintaining the gut epithelial barrier. So it has been hypothesized that microbiome dysbiosis will negatively affect the microbial barrier and will also adversely affect the epithelium by reducing butyrate levels and increasing cell permeability (Earley et al., 2015).

Dysbiosis of the gastrointestinal microbiome after a burn injury has been reported in a few studies (Earley et al., 2015; Shimizu et al., 2015), but some limitations are noteworthy. First, a large number of samples from healthy donors were used as the control group, and the gastrointestinal microbiome is hugely diverse among healthy individuals as revealed by the Human Microbiome Project (Human Microbiome Project Consortium, 2012b). Second, most burns patients are given antibiotics after their hospitalization and the gastrointestinal microbiome will be influenced by these treatments making it hard to distinguish which factor (burns or antibiotics) led to the microbiome disturbance (Mikkelsen et al., 2015; Tulstrup et al., 2015).

**FIGURE 1:**
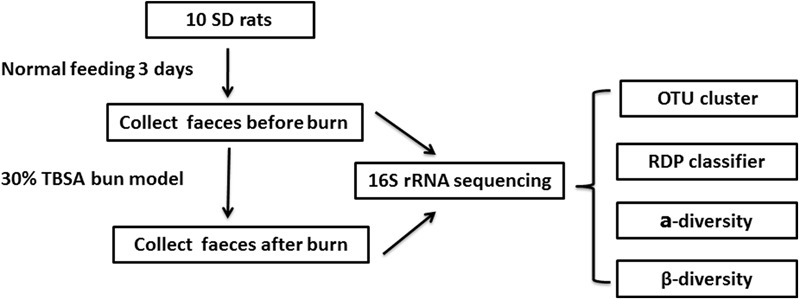
Diagrammatic sketch of the experimental protocol.

To investigate whether burn trauma affects the gastrointestinal microbiome in the early stages after the trauma, we collected fecal samples from individual animals before and after burn injury and analyzed the samples using16S rRNA sequencing (**Figure 1**). We found that the total number of species in the gastrointestinal microbiome did not change in the early stage after a burn injury. However, the ratio of some microbiomial components changed at the level of Phylum, Class, Order, Family and Genus.

## Materials and Methods

### Ethics Statement

This study was approved by the ethics committee at Southwest Hospital of Third Military Medical University, China. Healthy adult male Sprague-Dawley (SD) rats (*n* = 10) were purchased from the Animal Center of Daping Hospital of Third Military Medical University, China.

### Rat Burn Model and Sample Collection

Nine-week-old SD rats (180–220 g) were housed for 3 days under pathogen-free conditions with food and water provided *ad libitum*. The SD rats (10) were fed in 10 different cages. The gauzes (for bedding) were changed every day for cleanliness. Feces were collected for 3 days initially, before the burn model reported previously (Xiao et al., 2012) was applied. The rats were anesthetized with 1 g/L of pentobarbital sodium (30 mg/kg) injected intraperitoneally. A third degree burn covering 30% total body surface area (TBSA) was made by scalding the nude skin in a 96°C water bath for 25 s. Adequate measures were taken to minimize pain in the experimental animals. Physiological saline was injected based on their weight to prevent shock and the dressing covers were changed every day to prevent wound infection. Feces were also collected twice a day for 3 days after the burn was inflicted (**Figure 1**) and animals were sacrificed by decapitation under pentobarbital sodium anesthesia. All the fecal samples were stored at -80°C immediately after collection.

### DNA Purification and Sequencing

For each rat, at least three fresh feces samples were collected before and after burn injury. Before DNA purification, the three samples were mixed together and a total of 200 mg of the sample was used for DNA purification. A PowerFecal DNA Isolation Kit (Promega) was used to purify the DNA from the samples. A Qubit 2.0 was used to quantify the DNA before PCR amplification. 16S rRNA gene primers V3 and V4 (Claesson et al., 2010) (341F, 5′- CCTACGGGNGGCWGCAG-3′ and 805R, 5′- GACTACHVGGGTATCTAATCC-3′) were used for DNA sequencing. PCR products were quantified with Qubit2.0 and DNA sequencing was done with Illumina Miseq 2 × 300 (Sangon, China).

### Bioinformatics Analysis

#### Operational Taxonomic Unit (OTU) Analysis

UCLUST (Edgar, 2010) was used to identify the OTUs. The longest reads were selected as the target sequences and these were compared with the remaining reads. Reads with similarities above 0.97 were classified as OTUs. By the same process, all the reads were classified into different OTUs. The OTUs were considered to approximate to the genus level.

#### α-Diversity Analysis

The following indexes were used to investigate α-diversity (Human Microbiome Project Consortium, 2012b) in the samples: Coverage, richness index, Shannon index, ACE index, and Chao 1 index. Coverage is the average number of times that each nucleotide is expected to be sequenced given a certain number of reads (Sims et al., 2014). High coverage indicated a low level of missing information. The richness index represents the number of species. Higher richness index indicates more species in that sample. The Shannon index was used to investigate heterogeneity in the microbiome. The ACE index and Chao 1 index show the total number of species via use of two different formulas. Chao1 index is useful for data sets skewed toward the low-abundance classes, as in microbes (Hughes et al., 2001; Durden and Dong, 2009). The ACE index incorporates data from all species with fewer than 10 individuals, rather than just singletons and doubletons (Hughes et al., 2001). All the above α-diversity indexes were calculated using Mothur (Schloss et al., 2009).

#### β-Diversity Analysis

The following two indexes were used to assess β-diversity (Mikkelsen et al., 2015) in the samples: clustering analysis and Principal Component Analysis (PCA). They are two most commonly used indexes of β-diversity analysis. Clustering analysis can group a set of objects. In this study, post-burn samples would show a tendency to cluster together if burn injury has a certain effect on gut microbiota. PCA 3d plot could display the relative position of each sample in three dimensions. These two analyses were based on the UniFrac (Lozupone and Knight, 2005), which was used to calculate the species composition similarities among the samples. First, every OTU target sequence was aligned to the Greengenes database (DeSantis et al., 2006). Next, a phylogenetic tree based on the sequence alignments was acquired. Sample distances were calculated based on the sum of the unique branch lengths via UniFrac. Dendrograms represent hierarchical clustering of samples. PCA was used mainly to analyze the bacterial composition differences among the samples.

### Statistical Analysis

Differences between pre- and post-burn groups were determined by paired Student’s *t*-test using GraphPad Prism version 5 for Windows (GraphPad Software, La Jolla, CA, United States^1^). Differences were considered significant when *p* < 0.05.

## Results

### Sample Sequencing

DNA was extracted from 10 pre-burn fecal samples and 10 post-burn fecal samples. PCR and agarose gel electrophoresis showed that the 16S rRNA gene was successfully amplified (**Supplementary Figure S1**). After sequencing, an average of 13614 reliable reads was obtained for each sample with a mean length of 416-bp (**Table 1**). The read number for each sample exceeded 10000 (range 10103 to 16370), thus indicating good sequencing coverage of the samples.

**Table 1:** 16S rRNA gene sequencing statistics for the 20 pre- and post-burn samples.

Sample_name	Barcode	Raw_num	Mean_len	Clean_num	Mean_len
BurnB1	CTCTCTG	14621	450.8	14620	412.4
BurnB2	TCTCGTC	13694	452	13692	413.6
BurnB3	AGCTGAC	14746	455.1	14743	416.7
BurnB4	CACTAGA	14702	452.4	14699	414
BurnB5	CACTCAG	15107	453.8	15104	415.5
BurnB6	TCGATAC	12942	452.7	12939	414.4
BurnB7	CATCACG	15774	449.3	15773	410.9
BurnB8	CGATATG	14448	455.8	14444	417.6
BurnB9	CTACTAT	12739	455.1	12738	416.7
BurnB10	CGTCTGC	14921	456.7	14920	418.4
BurnA1	CGACGTC	14841	457.6	14839	419.3
BurnA2	ATGCGTA	10646	453.8	10646	415.5
BurnA3	TCATAGC	11135	455.8	11134	417.5
BurnA4	TCTATAG	10379	455.9	10379	417.6
BurnA5	TACGCAC	10103	454.9	10101	416.6
BurnA6	CATATCA	12040	455.1	12034	416.8
BurnA7	AGCGAGT	14255	457	14253	418.6
BurnA8	AGACTAC	16370	455.4	16367	417
BurnA9	TCGACTG	13934	454.7	13934	416.3
BurnA10	AGTACGA	14928	456.9	14926	418.6

*BurnB1 to BurnB10 denotes the 10 pre-burn samples. BurnA1 to BurnA10 denotes the 10 post-burn samples. Clean_num and Clean_len denote the number of reads and the read lengths after quality control.*

### OTU Analysis

An average of 2287.1+163.63 and 2416.5+155.70 OTUs (**Table 2**) were called with a similarity score of 0.97 for the pre- and post-burn groups, respectively. The statistical analysis showed no significant differences between the two groups. The burn injury did not affect the OTU count. This shows that the total number of species in the microbiome did not change significantly in the early stage (first 3 days) of the burn injury.

**Table 2:** Operational taxonomic unit (OTU) numbers before and after the burn injury.

Average num	Post-burn	Pre-burn
Reads	10578 ± 2475.79	12106 ± 1262.76
OTUs	2416.5 ± 155.70	2287.1 ± 163.63

*Statistical analysis based on paired *t*-tests. The *p*-values are 0.4683 and 0.1178 for the sequence reads and OTUs, respectively. The results were presented as “Mean ± standard deviation.”*

### α-Diversity

**FIGURE 2:**
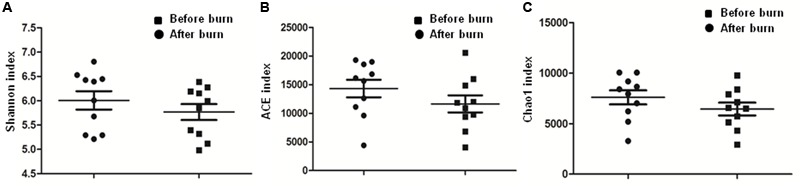
Comparison of Shannon index **(A)**, ACE index **(B)**, and Chao 1 index **(C)**. Paired student’s *t*-test was used for the statistical analysis and the *p*-value for each comparison is 0.3038, 0.0606, and 0.0619, respectively.

To calculate the percent sequence coverage, Good’s method (Good, 1953) was used with the [1-(*n*/*N*)] × 100 formula, where *N* represents the number of all the sequences and *n* represents the number count of the single-occurrence sequences. The average coverage of the 10 pre-burn samples came to 87% and the average coverage of the 10 post-burn samples came to 83% (**Supplementary Table S1**). As shown in **Figure 2** and **Table 3**, no significant differences were identified for all three α-diversity indexes (Shannon index, ACE index and Chao1 index). Also, no statistically significant differences were found in the rarefaction plots for richness, Shannon index, ACE index and Chao1 index (**Supplementary Figure S2**). These results show that the bacterial community diversity among the groups did not change in the early stage after the burn injury.

**Table 3:** α-diversity of the gastrointestinal microbiome in rats before and after experiencing a burn injury.

	Richness	Shannon	ACE	Chao1
	index	index	index	index
Post-burn	2416.5 + 492.36	6.01 + 0.60	14328 + 4825.54	7601.2 + 2189.93
Pre-burn	2287.1 + 517.43	5.77 + 0.52	11638 + 4702.10	6440.9 + 2016.58

*The results were presented as “Mean ± standard deviation.”*

### Sample Distance and Sample Clustering Analysis

**FIGURE 3:**
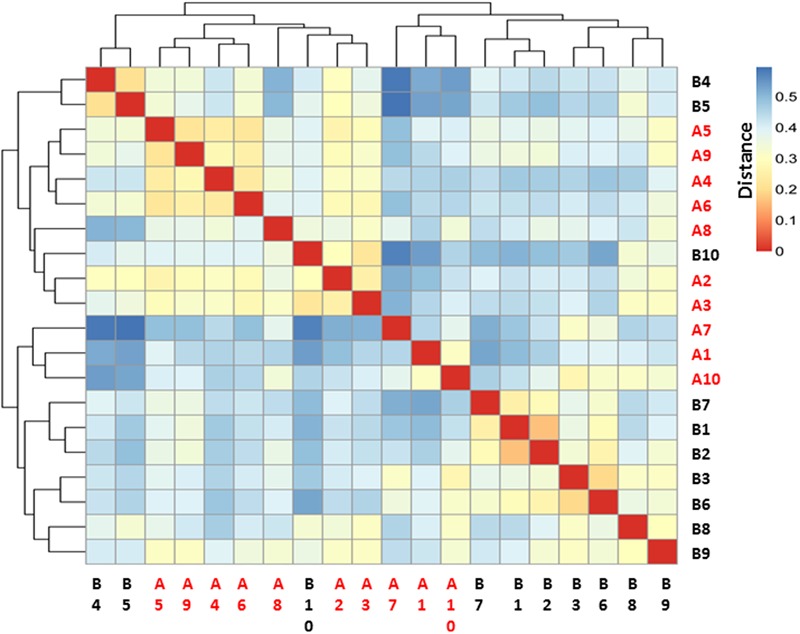
Heatmap for sample–sample distance. Red color indicates the nearest distance and blue color indicates long sample–sample distance. Pre-burn samples were named as B1–B10 in black and post-burn samples were named as A1–A10 in red.

The microbiome community relationships among the samples from the two groups (pre- and post-burn) is shown in **Figure 3**. It was expected that the 20 samples would cluster into 10 paired groups if the burn injury had no effect on the gastrointestinal microbiome based on the sample distance. As shown in **Figure 3**, B4, 5, 10 and A2, 3, 4, 5, 6, 8, 9 have shorter sample distances and group as one cluster, while the 10 remaining samples belong to another cluster. The majority (7/10) of the pre-burn samples grouped as one cluster, while another cluster consisted mainly of the post-burn samples (7/10). This indicates that the burn injury influenced the gastrointestinal microbiome in the rats.

**FIGURE 4:**
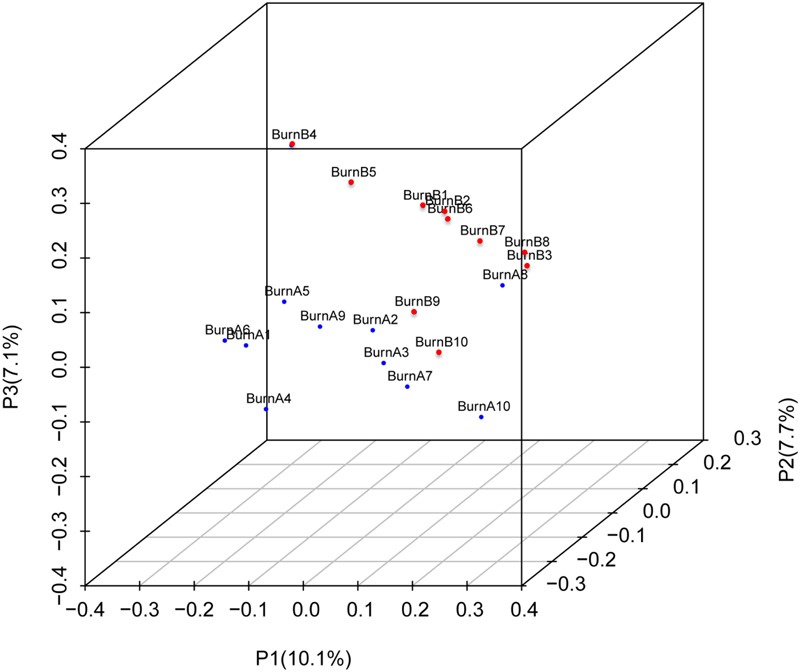
Principal component analysis (PCA) 3D plot of the operational taxonomic unit. Pre-burn samples are shown in blue and the post-burn samples are shown in red. PCA axis 1 accounts for 29.8% of the inter-sample variation, PCA axis 2 accounts for 18.7% of the inter-sample variation, and PCA axis 3 accounts for 13.2% of the inter-sample variation.

**Figure 4** shows the PCA results for the 10 pre-burn samples (in blue) and 10 post-burn samples (in red). After the burn injury, the fecal samples tended to gather together (**Figure 4**). The samples showed more potential to cluster on P2 and P3, which accounted for 18.7 and 13.2% of the inter-sample variation, respectively. The PCA of these data indicated that community composition differences exist between the samples collected before and after the burn injury. This also indicates that burn trauma disturbed the gastrointestinal microbiome.

### Ribosomal Database Project (RDP) Classifier

**FIGURE 5:**
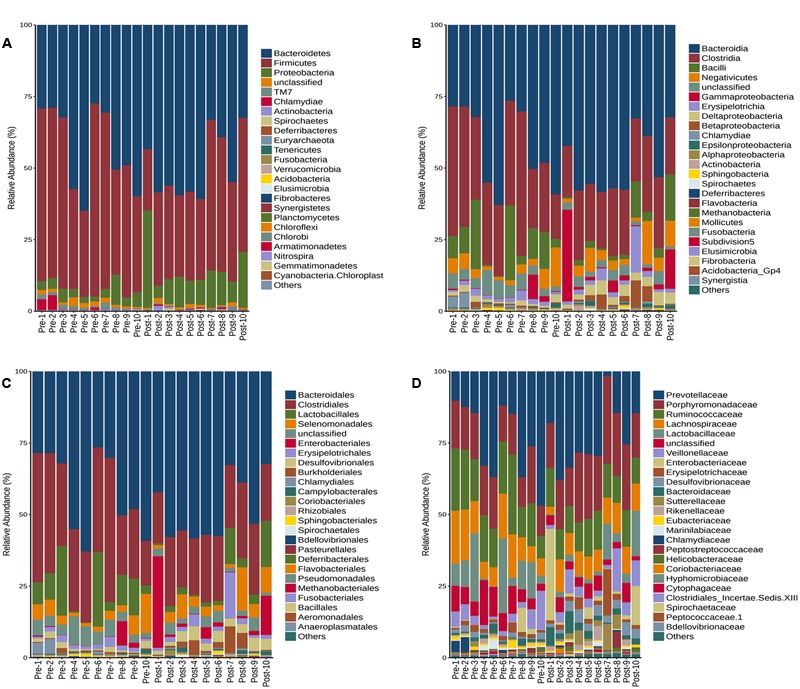
Taxonomic assignments of the bacterial tags from the two groups (pre- and post-burn) in Phylum **(A)**, Class level **(B)**, Order **(C)**, and Family level **(D)**. BurnB1 denotes the result for rat number 1 pre-burn, while BurnA1 denotes the result for rat number 2 post-burn and so on up to BurnB10 and BurnA10. The bar indicates the bacterial classification at each level.

The RDP classifier was used to classify the sequence reads at the phylum to genus level. In most of the rats, the dominant bacteria remained unchanged after the burn trauma. However, the ratio of some bacteria decreased significantly at different levels after the burn injury (**Supplementary Table S2**). The number of phyla in the two groups did not differ (**Supplementary Figure S3**). On average, 13 and 14 phyla were identified in each sample before and after the burn injury, respectively. Firmicutes and Bacteroidetes were the two dominant phyla in this animal model, and they comprised about 92.0% of the pre-burn group. However, these two dominant phyla decreased to 85.2% after the burn injury. Concurrently, the Proteobacteria levels increased from 4 to 12.6% after the burn injury (**Figure 5A** and **Supplementary Table S2**). The Firmicutes/Bacteroidetes ratio decreased significantly after the burn injury (**Supplementary Figure S4**). At the class level (**Figure 5B**), the ratio of Clostridia (khaki color) decreased from 31.7 to 22.1%. And at the order level (**Figure 5C**), Desulfovibrionales increased significantly from 1.2 to 3.2%. The level of Clostridiales reduced from 31.7 to 22.1%. At the family level (**Figure 5D**), Desulfovibrionaceae, Enterobacteriales and Porphyromonadaceae increased from 1.2, 1.2, and 15.1% to 3.1, 5.6, and 20.3%. Increases of Enterobacteriaceae post-severe burn was also reported by Earley et al. (2015). They also found the evidence of Enterobacteriaceae translocation using fluorescence *in situ* hybridization method (Earley et al., 2015).

**FIGURE 6:**
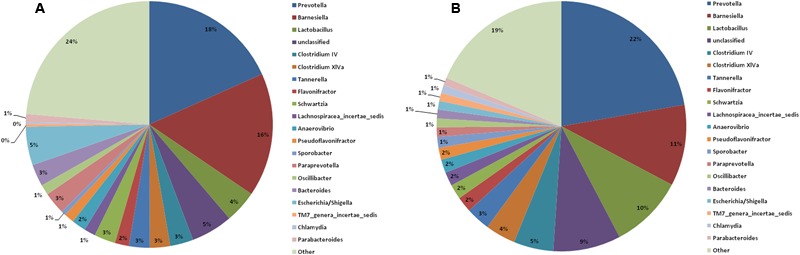
Constituents of the gastrointestinal microbiome before **(B)** and after **(A)** burn injury at the genus level. Each genus is indicated by different colors or by other means (light green) for all other genera not in the top-20 list.

At the genus level, the top 20 genera in the two groups are shown in **Figure 6**. The remaining genera were combined and are shown as “other” in the figure. The Lactobacillus abundance decreased from 9.6% before the burn injury to 4.3% after the burn injury (**Supplementary Table S3**). A previous study reported that Lactobacillus is a potential probiotic. The functions performed by Lactobacillus include maintaining the microbiome balance, regulating the immune system, and enhancing the metabiotic capacity of the gut. *Escherichia* and *Shigella* are known opportunistic pathogens in the gastrointestinal tract. The abundance of these potentially pathogens increased from 1.1 to 5.0% after the burn injury (**Figure 6**). Except for these two genera, the ratios of many other genera changed after the burn injury. For example, Bilophila increased from 0.6 to 2.2% after burn injury, while Barnesiella increased from 10.5 to 16.2% (**Supplementary Table S2**). In contrast, Clostridium IV decreased from 5.1 to 3.0% after the burn injury. The abundance of Clostridium XlVa also decreased from 4 to 3%. It is known that both Clostridium IV and Clostridium XlVa are butyrate-producing bacteria (Van den Abbeele et al., 2013).

## Discussion

Infection is considered as the primary cause of death in burn patients. Burn injury not only damages the integrity of skin barrier, but also interrupts the immune status of human body. Apidianakis et al. (2012) found that hGSTA4 down-regulation lead to overexpression of lipid peroxidation byproduct 4HNE post-severe burn. And that finally leads to high susceptibility to bacterial (especially *Pseudomonas aeruginosa*) infections (Apidianakis et al., 2012).

The gastrointestinal tract is a place where the most abundant microorganisms exist in the body, especially bacteria (Human Microbiome Project Consortium, 2012b; Sommer and Backhed, 2013). This microbial system is relatively stable unless it is disrupted by antibiotics or disease (Sommer and Backhed, 2013; Mikkelsen et al., 2015; Tulstrup et al., 2015), and provides benefits to the host by producing nutrition directly and indirect pro-immune defense functions (Yurist-Doutsch et al., 2014). The system also plays an important role in many health-related issues and diseases, such as obesity, inflammatory bowel disease, and asthma. The functions of the gastrointestinal microbiome and its dysbiosis after trauma were elucidated recently. 16S rRNA deep sequencing is the main method used for comparing differences in the microbiome community in various settings. One advantage of 16S rRNA analysis is that it can accurately determine the microbial composition of a test sample without the need for *in vitro* culturing (Claesson et al., 2010; Wu et al., 2011). The high sensitivity of 16S rRNA sequencing means that underrepresented bacterial groups can be detected with the method, even when only a few colonies are present in a sample. Because we collected the samples for 3 days after the burn injury, the short period may not sufficient for a species to vanish completely. Therefore, it seems reasonable that the total number of OTUs in the gastrointestinal microbiome did not change significantly in our study. Based on the results of the Human Microbiome Project (Human Microbiome Project Consortium, 2012a,b), it is possible that some species might vanish completely through medical practices and lifestyle changes (Blaser and Falkow, 2009).

Firmicutes comprise mostly Gram-positive bacteria with a DNA content of low G+C, but they also include Gram-negative bacteria. Bacteroidetes include Gram-negative bacteria, which are represented mainly by the Bacteroides genus in the human gut. Proteobacteria consist mainly of Gram-negative bacteria and include a wide variety of well-studied pathogens. The Proteobacteria abundance increased significantly after the burn injury in our study (**Figure 5A**). The ratio of Firmicutes/Bacteroidetes has been shown to be of significant relevance for signaling human gut microbiota status determined by 16S rRNA sequencing and species-specific quantitative PCR (Ley et al., 2006; Mariat et al., 2009). The Firmicutes/Bacteroidetes ratio decreased significantly in the early stage after the burn injury in our study.

Dysbiosis of the gastrointestinal microbiome has two aspects to it. First, the abundance of some probiotic microbes decreased after the burn injury. Butyrate, a short-chain fatty acid, provides benefits to the epithelium, but butyrate-producing bacteria decreased significantly after the burn injury. According to another study by Kuethe et al. (2016), they also found that burn trauma would lead to significant decrease in the butyrate producing bacteria *R. gnavus, C. eutactus*, and *Roseburia* species. Shimizu et al. (2015) also found that butyric acids decreased to lower-than-normal levels among five serve burn patients. Inconsistent with Shimizu’s finding, we did not found any increases of *Pseudomonas* and *Candida* with rat model. The pathogenic bacteria in this study are *Escherichia* and *Shigella.*

Lactobacillus, which also decreased significantly, plays an important role in maintaining the balance of the gastrointestinal microbiome. Second, the abundance of some potentially pathogenic bacteria increased after the burn injury. Proteobacteria, *Escherichia* and *Shigella* levels clearly increased after the burn injury (**Figures 5A, 6**); these genera consist of many opportunistic pathogens. Many conditional pathogens might proliferate quickly during microbiome dysbiosis. Furthermore, the micro-environment of the gastrointestinal tract could change significantly following burn injury and this might lead to an immunity disorder.

The present study has some limitations. First, the influence of the anesthesia or manipulation of the rats could not be evaluated in terms of the effect they might have had on the microbiome. A sham control and larger sample sizes in the burn injury group and control group would help resolve this problem. Second, the luminal microbiota and the mucosal-associated microbiota are two different communities (Ringel et al., 2015), and we do not know how the microbiome changes exactly in the mucosa.

## Conclusion

We used a rat model to evaluate the effect of burn injury on the gastrointestinal microbiome of these animals. The gastrointestinal microbial community changed after the burn injury. Although the total number of OTUs did not change significantly, the constituents of the microbiome community did. The abundance of probiotic organisms decreased after the burn injury, while the abundance of opportunistic organisms increased. More research is needed to determine whether a relationship between microbiome disturbance and clinical illness and clinical outcomes exists.

## Author Contributions

YP, ZY, and JC conceived and designed this study. KS, SY, YC, and BJ carried out the experiments. GH, YG, YC, ZY, and KS analyzed the data. GH and YP drafted the manuscript. All authors have read and approved the final manuscript.

## Conflict of Interest Statement

The authors declare that the research was conducted in the absence of any commercial or financial relationships that could be construed as a potential conflict of interest.

## Footnotes

1 www.graphpad.com

## Supplementary Material

FIGURE S1 16S rRNA PCR results for the 20 samples.

FIGURE S2 Rarefaction analysis of each sample from the two groups. The *X*-axis represents the number of sequences and the *Y*-axis represents the Shannon index **(A)**, number of operational taxonomic units **(B)**, Chao1 index **(C)**, and ACE index **(D)**.

FIGURE S3 Comparison of the number of phyla in the two groups. A paired *t*-test was used for the statistical analysis and the *p*-value obtained was 0.4642.

FIGURE S4 Firmicutes/Bacteroidetes ratio comparison of the two groups. ^∗^*p* < 0.05.

TABLE S1 α-diversity indexes for each sample.

TABLE S2 ALL the phyla, classes, orders, families, and genera that changed in this study.

TABLE S3 All samples at genus level.

## References

[B1] Apidianakis Y. Que Y. A. Xu W. Tegos G. P. Zimniak P. Hamblin M. R. (2012). Down-regulation of *glutatione S-transferase alpha 4* (hGSTA4) in the muscle of thermally injured patients is indicative of susceptibility to bacterial infection. *FASEB J.* 26 730–737. 10.1096/fj.11-192484 22038048 PMC3290433

[B2] Barcenilla A. Pryde S. E. Martin J. C. Duncan S. H. Stewart C. S. Henderson C. (2000). Phylogenetic relationships of butyrate-producing bacteria from the human gut. *Appl. Environ. Microbiol.* 66 1654–1661. 10.1128/AEM.66.4.1654-1661.2000 10742256 PMC92037

[B3] Blaser M. J. Falkow S. (2009). What are the consequences of the disappearing human microbiota? *Nat. Rev. Microbiol.* 7 887–894. 10.1038/nrmicro2245 19898491 PMC9354563

[B4] Claesson M. J. Wang Q. O’Sullivan O. Greene-Diniz R. Cole J. R. Ross R. P. (2010). Comparison of two next-generation sequencing technologies for resolving highly complex microbiota composition using tandem variable 16S rRNA gene regions. *Nucleic Acids Res.* 38 e200 10.1093/nar/gkq873 PMC3001100 20880993

[B5] Collins M. D. Lawson P. A. Willems A. Cordoba J. J. Fernandez-Garayzabal J. Garcia P. (1994). The phylogeny of the genus *Clostridium*: proposal of five new genera and eleven new species combinations. *Int. J. Syst. Bacteriol.* 44 812–826. 10.1099/00207713-44-4-812 7981107

[B6] DeSantis T. Z. Hugenholtz P. Larsen N. Rojas M. Brodie E. L. Keller K. (2006). Greengenes, a chimera-checked 16S rRNA gene database and workbench compatible with ARB. *Appl. Environ. Microbiol.* 72 5069–5072. 10.1128/AEM.03006-05 16820507 PMC1489311

[B7] Durden C. Dong Q. (2009). RICHEST–a web server for richness estimation in biological data. *Bioinformation* 3 296–298. 10.6026/97320630003296 19293995 PMC2655047

[B8] Earley Z. M. Akhtar S. Green S. J. Naqib A. Khan O. Cannon A. R. (2015). Burn injury alters the intestinal microbiome and increases gut permeability and bacterial translocation. *PLoS ONE* 10:e0129996 10.1371/journal.pone.0129996 PMC4496078 26154283

[B9] Edgar R. C. (2010). Search and clustering orders of magnitude faster than BLAST. *Bioinformatics* 26 2460–2461. 10.1093/bioinformatics/btq461 20709691

[B10] Fujimura K. E. Slusher N. A. Cabana M. D. Lynch S. V. (2010). Role of the gut microbiota in defining human health. *Expert Rev. Antiinfect. Ther.* 8 435–454. 10.1586/eri.10.14 PMC2881665 20377338

[B11] Good I. J. (1953). The population frequencies of species and the estimation of population parameters. *Biometrika* 40 237–264. 10.1093/biomet/40.3-4.237

[B12] Harris J. K. El Kasmi K. C. Anderson A. L. Devereaux M. W. Fillon S. A. Robertson C. E. (2014). Specific microbiome changes in a mouse model of parenteral nutrition associated liver injury and intestinal inflammation. *PLoS ONE* 9:e110396 10.1371/journal.pone.0110396 PMC4203793 25329595

[B13] Horai R. Zárate-Bladés C. R. Dillenburg-Pilla P. Chen J. Kielczewski J. L. Silver P. B. (2015). Microbiota-dependent activation of an autoreactive T cell receptor provokes autoimmunity in an immunologically privileged site. *Immunity* 43 343–353. 10.1016/j.immuni.2015.07.014 26287682 PMC4544742

[B14] Hughes J. B. Hellmann J. J. Ricketts T. H. Bohannan B. J. (2001). Counting the uncountable: statistical approaches to estimating microbial diversity. *Appl. Environ. Microbiol.* 67 4399–4406. 10.1128/AEM.67.10.4399-4406.2001 11571135 PMC93182

[B15] Human Microbiome Project Consortium (2012a). A framework for human microbiome research. *Nature* 486 215–221. 10.1038/nature11209 22699610 PMC3377744

[B16] Human Microbiome Project Consortium (2012b). Structure, function and diversity of the healthy human microbiome. *Nature* 486 207–214. 10.1038/nature11234 22699609 PMC3564958

[B17] Kuethe J. W. Armocida S. M. Midura E. F. Rice T. C. Hildeman D. A. Healy D. P. (2016). Fecal microbiota transplant restores mucosal integrity in a murine model of burn injury. *Shock* 45 647–652. 10.1097/SHK.0000000000000551 26682948 PMC5103310

[B18] Ley R. E. Turnbaugh P. J. Klein S. Gordon J. I. (2006). Microbial ecology: human gut microbes associated with obesity. *Nature* 444 1022–1023. 10.1038/4441022a 17183309

[B19] Lozupone C. Knight R. (2005). UniFrac: a new phylogenetic method for comparing microbial communities. *Appl. Environ. Microbiol.* 71 8228–8235. 10.1128/AEM.71.12.8228-8235.2005 16332807 PMC1317376

[B20] Mariat D. Firmesse O. Levenez F. Guimarǎes V. Sokol H. Doré J. (2009). The Firmicutes/Bacteroidetes ratio of the human microbiota changes with age. *BMC Microbiol.* 9:123 10.1186/1471-2180-9-123 PMC2702274 19508720

[B21] Mikkelsen K. H. Frost M. Bahl M. I. Licht T. R. Jensen U. S. Rosenberg J. (2015). Effect of antibiotics on gut microbiota, gut hormones and glucose metabolism. *PLoS ONE* 10:e0142352 10.1371/journal.pone.0142352 PMC4643023 26562532

[B22] Ringel Y. Maharshak N. Ringel-Kulka T. Wolber E. A. Sartor R. B. Carroll I. M. (2015). High throughput sequencing reveals distinct microbial populations within the mucosal and luminal niches in healthy individuals. *Gut Microbes* 6 173–181. 10.1080/19490976.2015.1044711 25915459 PMC4615648

[B23] Scheppach W. Bartram H. P. Richter F. (1995). Role of short-chain fatty acids in the prevention of colorectal cancer. *Eur. J. Cancer* 31A 1077–1080. 10.1016/0959-8049(95)00165-F 7576995

[B24] Schloss P. D. Westcott S. L. Ryabin T. Hall J. R. Hartmann M. Hollister E. B. (2009). Introducing mothur: open-source, platform-independent, community-supported software for describing and comparing microbial communities. *Appl. Environ. Microbiol.* 75 7537–7541. 10.1128/AEM.01541-09 19801464 PMC2786419

[B25] Sekirov I. Russell S. L. Antunes L. C. Finlay B. B. (2010). Gut microbiota in health and disease. *Physiol. Rev.* 90 859–904. 10.1152/physrev.00045.2009 20664075

[B26] Shimizu K. Ogura H. Asahara T. Nomoto K. Matsushima A. Hayakawa K. (2015). Gut microbiota and environment in patients with major burns - a preliminary report. *Burns* 41 e28–e33. 10.1016/j.burns.2014.10.019 25465986

[B27] Sims D. Sudbery I. Ilott N. E. Heger A. Ponting C. P. (2014). Sequencing depth and coverage: key considerations in genomic analyses. *Nat. Rev. Genet.* 15 121–132. 10.1038/nrg3642 24434847

[B28] Sommer F. Backhed F. (2013). The gut microbiota–masters of host development and physiology. *Nat. Rev. Microbiol.* 11 227–238. 10.1038/nrmicro2974 23435359

[B29] Tulstrup M. V. Christensen E. G. Carvalho V. Linninge C. Ahrné S. Højberg O. (2015). Antibiotic treatment affects intestinal permeability and gut microbial composition in Wistar rats dependent on antibiotic class. *PLoS ONE* 10:e0144854 10.1371/journal.pone.0144854 PMC4686753 26691591

[B30] Van den Abbeele P. Belzer C. Goossens M. Kleerebezem M. De Vos W. M. Thas O. (2013). Butyrate-producing *Clostridium* cluster XIVa species specifically colonize mucins in an *in vitro* gut model. *ISME J.* 7 949–961. 10.1038/ismej.2012.158 23235287 PMC3635240

[B31] Willems A. Amat-Marco M. Collins M. D. (1996). Phylogenetic analysis of *Butyrivibrio* strains reveals three distinct groups of species within the *Clostridium* subphylum of the gram-positive bacteria. *Int. J. Syst. Bacteriol.* 46 195–199. 10.1099/00207713-46-1-195 8573495

[B32] Wu L. Wang H. Zhang Z. Lin R. Lin W. (2011). Comparative metaproteomic analysis on consecutively *Rehmannia glutinosa*-monocultured rhizosphere soil. *PLoS ONE* 6:e20611 10.1371/journal.pone.0020611 PMC3105091 21655235

[B33] Xiao R. Teng M. Zhang Q. Shi X. H. Huang Y. S. (2012). Myocardial autophagy after severe burn in rats. *PLoS ONE* 7:e39488 10.1371/journal.pone.0039488 PMC3387177 22768082

[B34] Yurist-Doutsch S. Arrieta M. C. Vogt S. L. Finlay B. B. (2014). Gastrointestinal microbiota-mediated control of enteric pathogens. *Annu. Rev. Genet.* 48 361–382. 10.1146/annurev-genet-120213-092421 25251855

