# Supplementary figures and images for: Burn Injury Leads to Increase in Relative Abundance of Opportunistic Pathogens in the Rat Gastrointestinal Microbiome
Source: Front Microbiol. 2017 Jul 6;8:1237. doi: 10.3389/fmicb.2017.01237 (PMC5498482; doi:10.3389/fmicb.2017.01237)

Figure S1. 16S rRNA PCR results of 20 samples

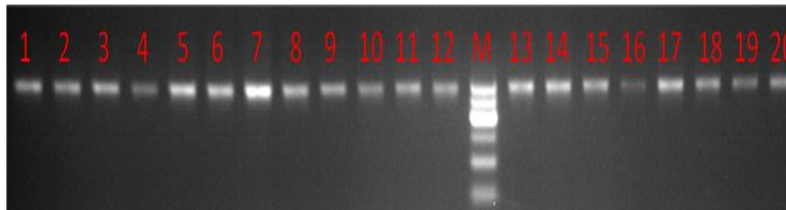

Supplement: FIGURE S1 — 16S rRNA PCR results for the 20 samples. [file Image_1.pdf]

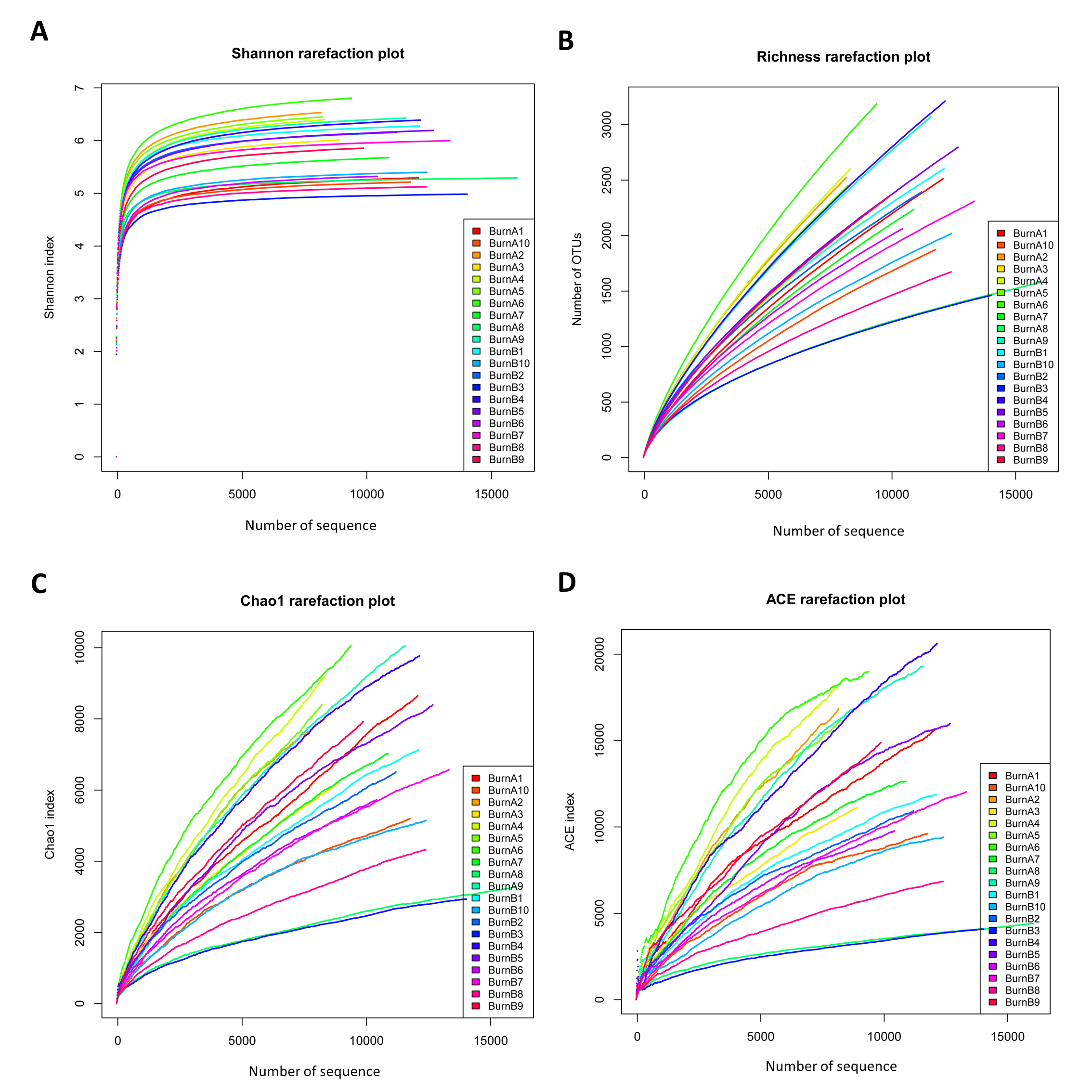

Supplement: FIGURE S2 — Rarefaction analysis of each sample from the two groups. The X-axis represents the number of sequences and the Y-axis represents the Shannon index (A), number of operational taxonomic units (B), Chao1 index (C), and ACE index (D). [file Image_2.TIF]

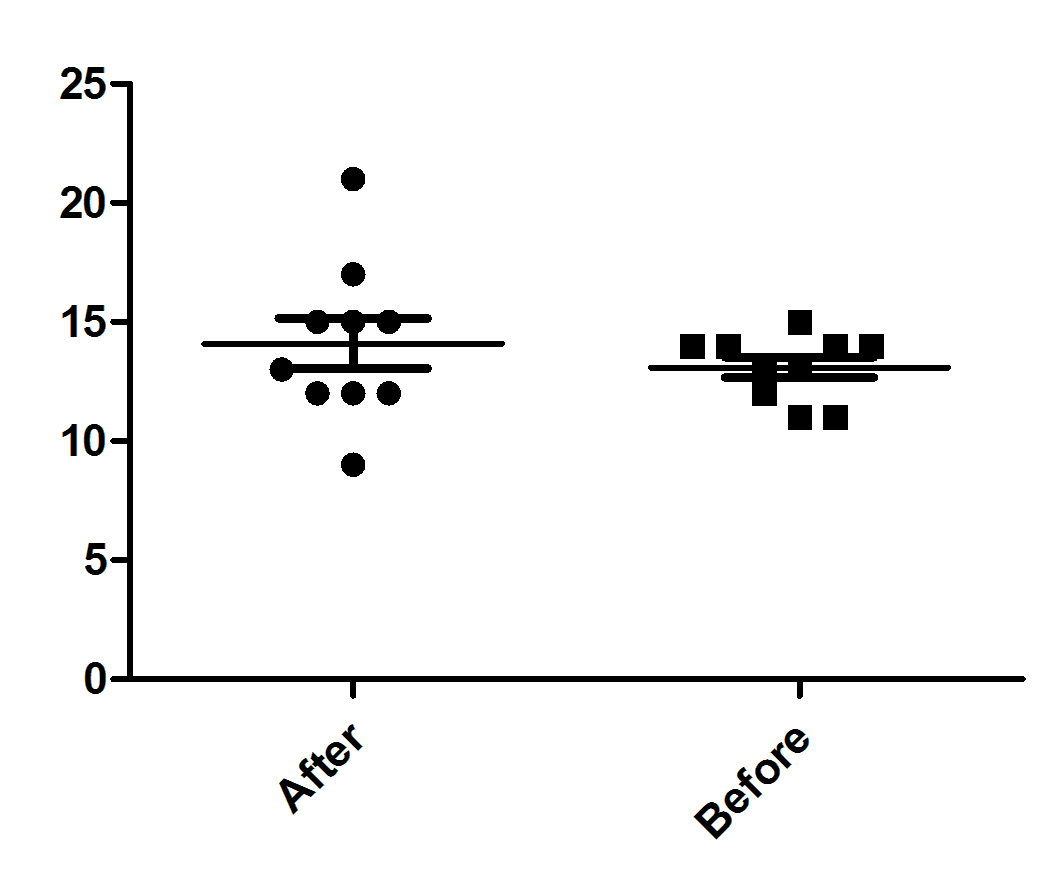

Supplement: FIGURE S3 — Comparison of the number of phyla in the two groups. A paired t-test was used for the statistical analysis and the p-value obtained was 0.4642. [file Image_3.TIF]

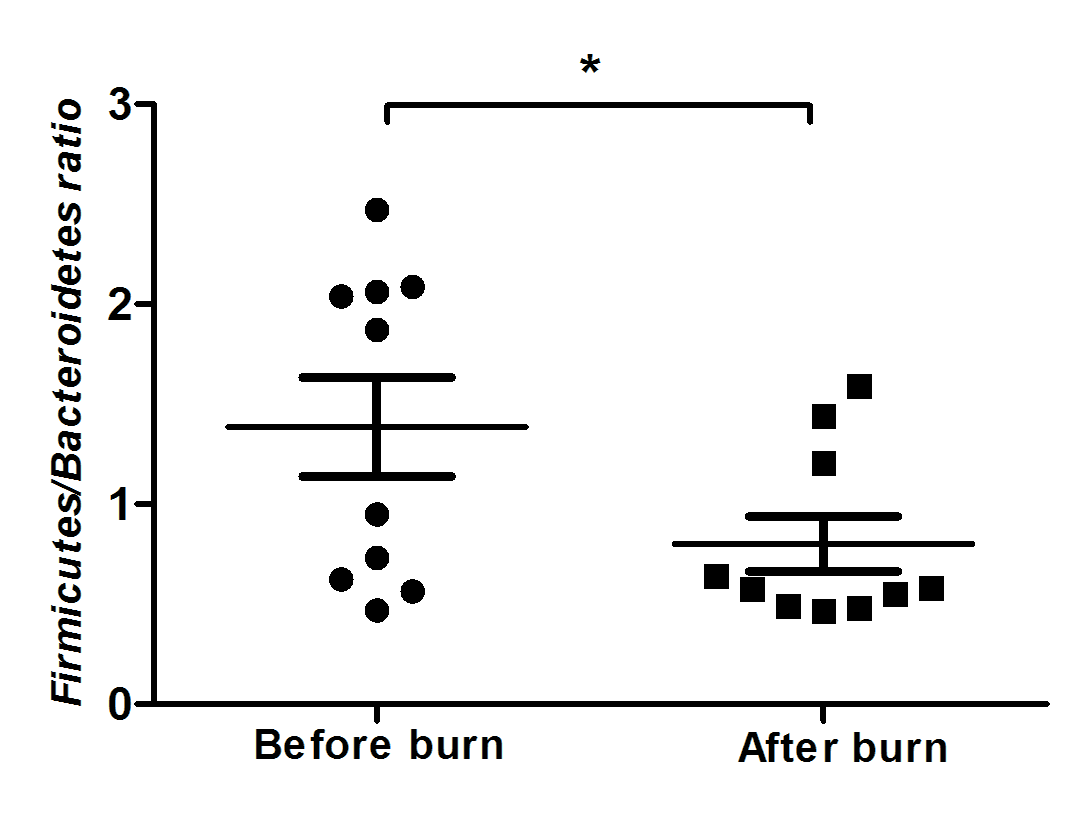

Supplement: FIGURE S4 — Firmicutes/Bacteroidetes ratio comparison of the two groups. ∗p < 0.05. [file Image_4.TIF]
