# Supplementary material for: Burn Injury Leads to Increase in Relative Abundance of Opportunistic Pathogens in the Rat Gastrointestinal Microbiome
Source: Front Microbiol. 2017 Jul 6;8:1237. doi: 10.3389/fmicb.2017.01237 (PMC5498482; doi:10.3389/fmicb.2017.01237)
Supplement: TABLE S1 — α-diversity indexes for each sample. [file Table_1.DOCX]

Figure S1. 16S rRNA PCR results of 20 samples


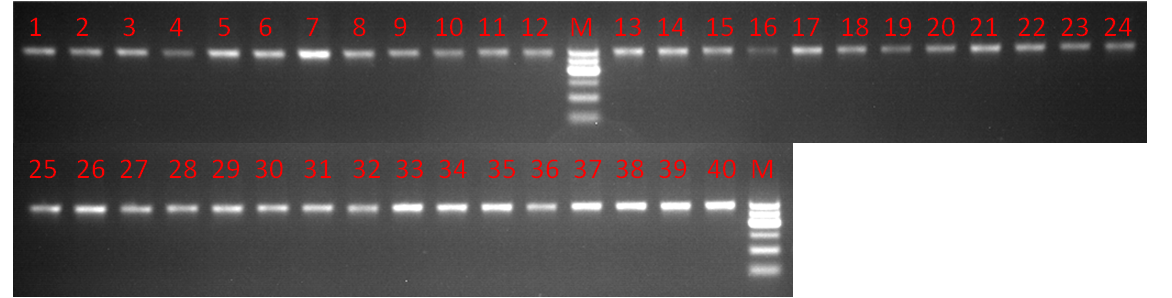


Table S1. α-diversity indexes of each sample

| Sample_ID | Seq_num | OTU_num | Shannon_index | ACE_index | Chao1_index | Coverage |
| --- | --- | --- | --- | --- | --- | --- |
| BurnA1 | 12116 | 2511 | 5.291597 | 15657.87 | 8652.552 | 0.852179 |
| BurnA10 | 11799 | 1872 | 5.211117 | 9607.346 | 5197.333 | 0.894228 |
| BurnA2 | 8209 | 2524 | 6.529315 | 16834.91 | 7939.01 | 0.780363 |
| BurnA3 | 8959 | 2137 | 6.007383 | 11119.88 | 6217.012 | 0.838933 |
| BurnA4 | 8366 | 2600 | 6.393387 | 18552.14 | 9177.772 | 0.772173 |
| BurnA5 | 8267 | 2450 | 6.446408 | 16154.64 | 8407.724 | 0.786622 |
| BurnA6 | 9425 | 3186 | 6.802607 | 18998.11 | 10059.01 | 0.757135 |
| BurnA7 | 10935 | 2235 | 5.675046 | 12637.35 | 7022.929 | 0.857613 |
| BurnA8 | 16081 | 1580 | 5.29076 | 4413.996 | 3278.266 | 0.947827 |
| BurnA9 | 11628 | 3070 | 6.425536 | 19308.66 | 10060.72 | 0.812178 |
| BurnB1 | 12161 | 2602 | 6.275534 | 11859.87 | 7134.525 | 0.860784 |
| BurnB10 | 12460 | 2018 | 5.397243 | 9395.873 | 5142.372 | 0.895907 |
| BurnB2 | 11242 | 2392 | 6.152658 | 10936.41 | 6508.817 | 0.861324 |
| BurnB3 | 14076 | 1462 | 4.982269 | 4080.617 | 2930.805 | 0.94551 |
| BurnB4 | 12195 | 3211 | 6.386821 | 20602.05 | 9767.326 | 0.81476 |
| BurnB5 | 12722 | 2796 | 6.190936 | 15982.52 | 8394.596 | 0.849552 |
| BurnB6 | 10470 | 2061 | 5.318952 | 9775.168 | 5726.522 | 0.870392 |
| BurnB7 | 13380 | 2309 | 5.9964 | 12030.34 | 6570.04 | 0.886173 |
| BurnB8 | 12441 | 1672 | 5.121357 | 6844.247 | 4315.866 | 0.916888 |
| BurnB9 | 9916 | 2348 | 5.854539 | 14874.04 | 7918.06 | 0.831989 |
