# Supplementary material for: Burn Injury Leads to Increase in Relative Abundance of Opportunistic Pathogens in the Rat Gastrointestinal Microbiome
Source: Front Microbiol. 2017 Jul 6;8:1237. doi: 10.3389/fmicb.2017.01237 (PMC5498482; doi:10.3389/fmicb.2017.01237)
Supplement: TABLE S3 — All samples at genus level. [file Data_Sheet_2.DOCX]

Table S2. ALL the phyla, classes, orders, families and genera that changed in this study

|  | Name | Post-burn. (%) | Pre-burn (%) | p-values |
| --- | --- | --- | --- | --- |
| phylum | TM7 | 0.354986 | 1.039043 | 0.010059 |
|  | Proteobacteria | 12.62645 | 4.002132 | 0.011539 |
|  | Firmicutes | 35.5626 | 49.015 | 0.025835 |
|  | Fusobacteria | 0.045998 | 0 | 0.034701 |
| class | Deltaproteobacteria | 3.296091 | 1.371058 | 0.000624 |
|  | Unclassified | 2.628154 | 4.545088 | 0.024965 |
|  | Clostridia | 22.14 | 31.71481 | 0.026905 |
|  | Fusobacteria | 0.046 | 0 | 0.034696 |
| order | Desulfovibrionales | 3.207234 | 1.256899 | 0.000878 |
|  | Clostridiales | 22.10587 | 31.67958 | 0.026795 |
|  | Fusobacteriales | 0.046 | 0 | 0.034684 |
|  | Unclassified | 2.811377 | 4.7737 | 0.036831 |
| family | Desulfovibrionaceae | 3.089196 | 1.197717 | 0.000785 |
|  | Porphyromonadaceae | 20.31073 | 15.05868 | 0.015203 |
|  | Ruminococcaceae | 11.34924 | 16.62709 | 0.019762 |
|  | Unclassified | 3.770544 | 7.259173 | 0.02489 |
|  | Clostridiales_Incertae Sedis XII | 0.003001 | 0.011997 | 0.027106 |
|  | Fusobacteriaceae | 0.045002 | 0 | 0.039396 |
|  | Staphylococcaceae | 0.014002 | 0.003999 | 0.046059 |
| genus | Lawsonia | 0.063994 | 0.015993 | 0.000918 |
|  | Bilophila | 2.22068 | 0.578699 | 0.001411 |
|  | Barnesiella | 16.2002 | 10.51186 | 0.006075 |
|  | Acetanaerobacterium | 0.260967 | 0.624723 | 0.007325 |
|  | Xylanibacter | 0.027006 | 0.003998 | 0.007326 |
|  | TM7_genera_incertae_sedis | 0.354921 | 1.038477 | 0.010079 |
|  | Dendrosporobacter | 0.004003 | 0.017994 | 0.018032 |
|  | Klebsiella | 0.196007 | 0.004999 | 0.019309 |
|  | Paraprevotella | 2.906297 | 1.239415 | 0.024136 |
|  | Ruminococcus | 0.362052 | 0.872685 | 0.024385 |
|  | Robinsoniella | 0.092988 | 0.320781 | 0.033527 |
|  | Acetivibrio | 0.090002 | 0.258837 | 0.03483 |
|  | Fusobacterium | 0.043993 | 0 | 0.041356 |
|  | Bulleidia | 0.001 | 0.007998 | 0.043527 |
|  | Clostridium IV | 3.040797 | 5.137897 | 0.049274 |
|  | Bacteroides | 2.872178 | 1.136234 | 0.049354 |

Weltch's t-test was used for statistical analysis.
